# Frequency Identification of Vibration Signals Using Video Camera Image Data

**DOI:** 10.3390/s121013871

**Published:** 2012-10-16

**Authors:** Yih-Nen Jeng, Chia-Hung Wu

**Affiliations:** Department of Aeronautics and Astronautics, National Cheng Kung University, Tainan 70701, Taiwan; E-Mail: p4895112@mail.ncku.edu.tw or datoefi@msn.com

**Keywords:** image data acquisition, vibration signal, insufficient frame rate, time frequency analysis

## Abstract

This study showed that an image data acquisition system connecting a high-speed camera or webcam to a notebook or personal computer (PC) can precisely capture most dominant modes of vibration signal, but may involve the non-physical modes induced by the insufficient frame rates. Using a simple model, frequencies of these modes are properly predicted and excluded. Two experimental designs, which involve using an LED light source and a vibration exciter, are proposed to demonstrate the performance. First, the original gray-level resolution of a video camera from, for instance, 0 to 256 levels, was enhanced by summing gray-level data of all pixels in a small region around the point of interest. The image signal was further enhanced by attaching a white paper sheet marked with a black line on the surface of the vibration system in operation to increase the gray-level resolution. Experimental results showed that the Prosilica CV640C CMOS high-speed camera has the critical frequency of inducing the false mode at 60 Hz, whereas that of the webcam is 7.8 Hz. Several factors were proven to have the effect of partially suppressing the non-physical modes, but they cannot eliminate them completely. Two examples, the prominent vibration modes of which are less than the associated critical frequencies, are examined to demonstrate the performances of the proposed systems. In general, the experimental data show that the non-contact type image data acquisition systems are potential tools for collecting the low-frequency vibration signal of a system.

## 1. Introduction

Because of the rapid development of Web sites and computer software and hardware, peripheral devices such as electret condenser microphones [1] and webcams are becoming increasingly popular. In addition, low-level high-speed digital video cameras with a price and frame speed of less than US$3,000 and 300 FPS, respectively, are also common products. Because all such peripheral devices are mass produced, they are often cheap yet still offer high quality. Thus, there is potential to develop a data acquisition system using a notebook or PC and one of these peripheral devices as a sensor. In a previous paper, we proposed such a system using an electret condenser microphone [1]. This is another work that proposes using image devices for practical applications. Because the image signal closely relates to human eyes, numerous related studies can be easily accessed on the Internet [2,3]. Most of these works considered the spatial details of the images [2–6]. In this study, the vibration signal of a system in operation was obtained from the images in an entirely distinct manner.

Suppose a video camera is properly focused to take a frame from a target. Any small region of a series of resulting image frames involves the following temporal information: intensity variations of all incident light rays reaching the point of interest, the surface motion of the target, and the motion of the camera [7–9]. These obtained image data are frequently composed of the medium, significant surface movements, and all of the prominent vibration signals. Small to infinitesimal signals cannot be properly resolved by using these image systems. Because the captured video signal may still be highly complex, extracting the embedded physical mechanism within the image frames is difficult. In addition, the image signal embedded in a series of image frames has the problem of a limited number of significant figures (effective digits) and the existence of the non-physical oscillatory modes. When the frame rate is not rapid enough to resolve a dominant sinusoidal mode, one or more false modes are induced by the non-constant ratio of the pixel circuit for converting the photo-charge of the photo-diode to the gray level data [10–14].

Generally, the data size of a series of image frames is large. For example, the total pixels of a 600 × 300 frame is 1.8 × 10^5^. A user with 100 frames should manage the storage and data transfer of 18 M pixels. As the resolution is improved and the frame number increases, the difficulty is markedly increased. For the sake of fast data input/output by using the Internet or another available tool, almost all commercial video cameras use 1 B to resolve a pixel. That is, a single pixel can resolve only 0 to 255 gray levels of red, green, and blue, separately [6–10]. Such a low degree of resolution is sufficient for the human eye to detect image information. However, the human eye's resolution is too low for resolving accurately the vibration information embedded in complex image signals. Several previous studies [7–9] have partially overcome this difficulty by accumulating the total gray levels of neighboring pixels around a location of interest (the details of which are described in a subsequent Section). Using the same approach, the image signal in a region of a target surface can be converted to continuously distributed time series data, thus losing the spatial resolution in the process.

One relevant finding [7–9] is that the effective frequency response range of the image signals is in the low-frequency region. The first reason is the interference of the previous mentioned non-physical modes. The second is that the variation of the image signal is too small to be detected by the camera without an image-enhancing device. In this study, we attempted to identify the critical triggering frequency of the non-physical modes and to enhance the image signal simultaneously. We believe that the accurate frequency response in the region, in which the frequency is lower than the critical value, of image data acquisition can thus be identified. This is essential because the spectrum in the low-frequency region is closely related to the damage of many systems in operation. For example, in rotor-stator problems [15,16], the sub-harmonic vibrations have been attributed to the non-perfect alignment between components of a system or to a constant side force. Previous studies have further indicated that the sub-harmonic components provide a means for potentially exciting the low-frequency components of an aircraft. Furthermore, the most valuable information about a human being is also located in the low-frequency region. Most appealing of all, an image data acquisition system can be a noncontact and remote sensing system. If such a system is equipped with fiber glass and a properly arranged optical system, it can be used, for example, in a damaged underground region or other dangerous environments. Therefore, it is worthwhile to develop an image data acquisition system based on commercial webcams and high-speed cameras.

Generally, continuous time series data may involve a trend, a periodic part, and noise. Instrumental data are often contaminated by a mean trend resulting from processes other than those of primary interest [17–18]. Before applying the spectral method, this embedded trend should be removed to prevent the resulting spectrum from being contaminated [7–9,19–21]. To examine the periodic part, the Fourier method is applied to problems in which wave components involve only fixed amplitudes and frequencies [22–23]. To resolve the time-dependent amplitude and frequency, the iterative Gaussian filter and modified Gabor transform [1,7–9,19,23–26] are used to remove the trend and perform time frequency analysis, respectively.

This paper is organized as follows: Section 2 presents the experimental design and theory. Section 3 introduces a brief discussion on the related tools of data analysis. The performance of the proposed method is shown in the results and discussion section. Finally, the conclusions are presented in the final section.

## 2. Experimental Design and Theory

### 2.1. Experimental Design

**Figure 1.**
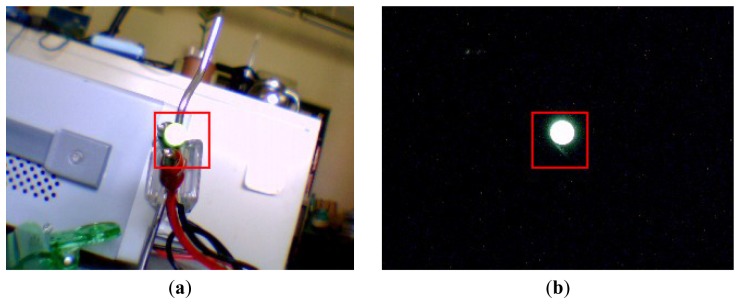
The test case of LED system: (**a**) the LED setup. (**b**) the luminescent state of LED system.

In this study, the image system involved uses a Prosilica CV640C CMOS high-speed camera with a 1394 interface and a Hawk 2.0 Megapixel webcam to obtain image data. The maximum frame rate of the high-speed camera is 500 FPS, with a resolution of 640 × 480, and those of the webcam are 35 FPS, with a frame resolution of 640 × 480. When the data were collected, their sampling rates were 230 and 30 FPS, respectively. First, the light signal emitted by a 5.0-mm Paralight L503GD green LED was used. The light source has the following specifications: the maximum power is 85 mW, the maximum voltage is DC 5 V, the mean current is 30 mA, and the peak current is 120 mA. The light source was powered by a function generator and was monitored using an oscilloscope. Figure 1(a,b) show a fraction of the experimental design, the image signal of which, emitted from the LED, was separately collected using the mentioned webcam and high-speed camera.

**Figure 2.**
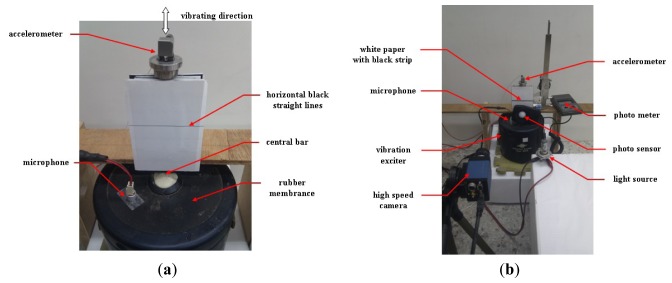
The experimental setup: (**a**) vibration exciter, accelerometer, and microphone. (**b**) overall setup.

To demonstrate further the capability of the high-speed camera and webcam image systems, the vibration signals emitted from an electrodynamics modal exciter type 4809 [9,19] were tested too. An accelerometer system and an acoustic data acquisition system with a small commercial microphone Figure 2(a,b) were also employed to validate the image data. The accelerometer is ceramic and has a resolution of 100 mV/g in a range from 0.5 to 3 kHz. The employed A/D card (NI USB·6210) has a 16-b resolution with a maximal sampling rate of 250 kb/s. The input signal to the exciter was obtained using an FG-350 function generator (IWATSU Electronic Co. Ltd) and monitored using an HP54603B oscilloscope. The data acquisition system was extended from that used in [1], who employed a small commercial microphone for personal and notebook computers to collect acoustic data. Specifications of the microphone are 20–20,000 Hz, 100 mW, 32 Ω, and 105 dB of sound pressure level sensitivity at 1 kZ ± 2%. As shown in [1], when the microphone is properly attached to the surface of the targeting system, the effective low-frequency bound can be extended from 20 Hz to approximately 0.5 Hz. The data conversion employed the built-in digital audio board and 16-b recording software of the Microsoft Windows XP operating system.

When data was collected from the design during the test periods, as shown in Figure 2(a,b), the vibration of the rubber membrane induced the up-and-down motion of the central bar. The input signal to the exciter was also obtained and monitored using the same function generator and oscilloscope, respectively. A white sheet of paper marked with a horizontal black line was attached to the bar. The black line, which was an output of a commercial laser printer, was printed horizontally to provide a large resolution of the vibration signal. In this study, varying line thicknesses were used, producing approximately one, three, five, seven, and nine effective pixels on the image frame by properly adjusting the distance between the paper and the high-speed camera (and/or the webcam). The accelerometer was located on top of the bar, and its sensor axis was positioned vertically with less than one degree of error. The microphone was attached to the rubber membrane of the exciter. To capture detailed image information using the high-speed camera, after turning off most light sources, the target was steadily shined using a properly powered car lamp to emit an illumination of 2,500 ± 125 LUX. The light intensity was monitored using a Lutron LX-101 digital photometer. The photometer has 1 LUX of resolution in the range of 0–1,999 LUX with ±5% error. In the ranges of 2,000–19,990 and 20,000–50,000 LUX, the resolutions are 10 and 100 LUX, respectively. The position of the car lamp was properly adjusted to emit different lumen data.

### 2.2. Image Data Properties

A webcam, which is a video camera that feeds its images in real time to a computer, can be a single object or a built-in component of a PC notebook [7–9]. Typically, a webcam includes an optical lens, an image sensor, and supporting electronics. The image sensor can be a CMOS active-pixel sensor or a charge-coupled device (CCD) [10–11]. Similarly, a high-speed camera also uses either a CMOS sensor or a CCD, which may record over 1,000 frames/s into its built-in DRAM [10–14].

In a CCD image sensor, reverse-biased p–n junctions (photodiodes) are used to absorb photons and produce charges representing sensed pixels, and the CCD is used to read these charges. An active-pixel sensor is another type of image sensor consisting of an integrated circuit containing an array of pixel sensors, with each pixel containing a photodetector and an active amplifier. For both CCD and CMOS image sensors, an image is projected through a lens onto the capacitor array of photodiodes or photodetectors, causing each capacitor to accumulate an electric charge proportional to the light intensity at that location. These charges are properly converted to voltages and are then sampled, digitized, and finally stored in memory [10–14].

For an incident ray of image to reach a photodiode or photodetector, a certain time interval is required so that the charge excited by the light reaches an equilibrium state. Subsequently, the charge also requires time to become a zero value so that the diode is active to the subsequently reached photons [11,13,14]. These intervals are closely related to the image lag and afterimage. To achieve all of the desired specifications, a typical pixel circuit is carefully attached to the photodetector, thus modifying these two time intervals. The resulting system is optimized to achieve a proper compromise among the following problems: image brightness and sharpness, desired frame speed, image lag, sensitivity, dynamic range, incomplete charge transfer, incomplete reset of charge, and noise [11,13,14]. The incomplete charge transfer and incomplete reset of charge frequently leave a residual charge in the pixel circuit. The residual charge eventually induces image persistence. Because most cameras are not designed to obtain the high-frequency spectrum of the vibration signal, how to completely avoid the generation of any non-physical sinusoidal response within the dynamic range is not a primary concern in most design processes. Consequently, most commercial video cameras have the problem of inducing a false mode when the frequency of a significant oscillatory mode embedded in the image signal exceeds a critical value. To detect the time-dependent information embedded in a series of frames, in an initial experiment, the mechanism of producing the non-physical response will be studied by a simple model in Section 2.4.

### 2.3. Enhancing Resolution of Image Data

The signal embedded in a single pixel of a target obtained using a digital high-speed camera may involve motions of the target surface and camera, combined with the lumen intensity variation of incident light. Fortunately, most significant modes of these signals are in different frequency bands and have low degrees of interference. In practical applications, if the discrete Fourier transform is a complete expansion, resolving details between different sources are not difficult. To suppress possible interference in this study, however, the camera was properly fixed, and the light intensity was properly controlled. Consequently, the information of the signal was dominated by the local motion of the target surface.

The surface motion of a system frequently involves many details such as the motion of the moving parts, the structural vibration of the system and components, and prominent acoustic signals. Consider a small local surface region of the system in two successive digital frames obtained using a high-speed camera. If the surface motion captured by a group of pixels in these films is adequately large, then the gray levels of these pixels certainly change. If their gray-level resolutions are fine enough, then the two-dimensional signals of the surface motion from pixel to pixel can be collected. Unfortunately, all of the pixels of most digital cameras have limited resolution, which is too rough to reflect most details, especially for small motions and/or images with insufficient contrast of brightness.

Generally, a solid surface has a certain degree of stiffness so that the corresponding surface motion is continuously and smoothly changed from point to point, except at the instance before cracking or failure. The following gray-level sum of pixels within a small square region [7–9] in a frame is thus a rough but reasonable approximation of the image data at the central point:

(1) $$\bar{g}=\sum_{i,jɛ\text{square}}g_{ij}$$

Although many shapes of the summed region can be used, to evaluate their sums and to locate their centers should require more computing time than that using a square. In practical applications, the size of the square should be carefully chosen. If the size is not large enough, the gray level resolution may be poor. On the other hand, if the square covers a region enclosing two or more components of the system, their vibration signals will be mixed together and are not easy to decouple them. In this study, all of the squares use a size of 50 × 50 pixels to achieve a proper compromise between these two restrictions.

If the surface movement is too small, then the summation may not change at all. Conversely, if the movement is too large, then it cannot consistently reflect most details of variation. Regarding the former case, a zoom lens or microscopic lens is helpful. For the latter case, increasing the distance between the target surface and the camera effectively reduces the negative effect.

**Figure 3.**
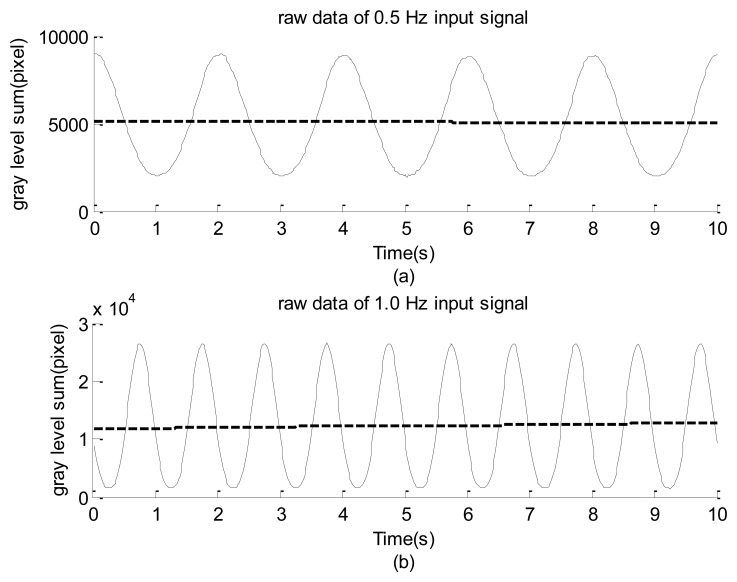
The LED image data of the high speed camera with 10.2 Hz input: solid line is the raw data and dashed line is the smooth part.

For example, consider the image shown in Figure 1(b) captured using a high-speed camera. The gray levels of the pixels within the region enclosed by the square (50 × 50 pixels) are summed using Equation (1) and are plotted with regard to time in Figure 3(a,b), in which the input signals were approximately 0.5 and 1.0 Hz, respectively. The reading from these figures shows that the maximal gray-level variations are approximately 7,000 and 24,000. These resolutions are much larger than 256. As mentioned in the introduction section, however, this approach has the drawback of losing the detailed spatial resolution of the surface of interest.

In practical applications, most target surfaces that reflect vibration signals do not emit light, such as the surface of the accelerometer shown in Figure 2(a,b). Consequently, the light intensity may not shine powerfully enough to actuate effectively the photodiode of a camera. To rescue the risk partially, in addition to providing an additional light source using a car lamp, the white paper marked with a straight black line shown in Figure 2(a,b) was employed to enhance the image signal. To reveal the effect, a series of frames similar to those shown in Figure 3(a,b) were examined.

**Figure 4.**
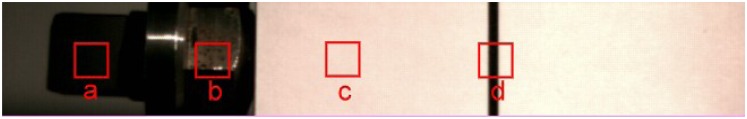
A strip type image of the high speed camera covers the accelerometer (involving two squares) and white paper sheet (involving another two squares).

**Figure 5.**
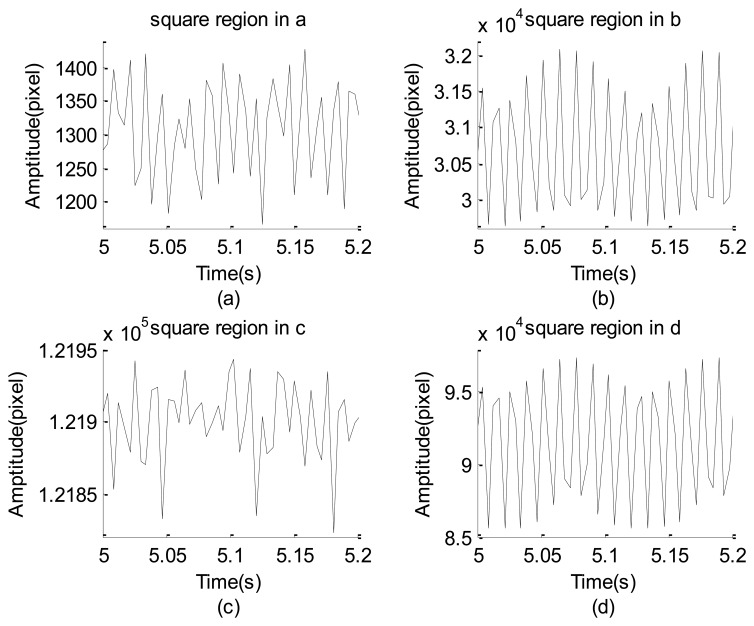
The gray level summation data of the four squares of Figure 2: (**a**) data of the leftmost square in a region of the accelerometer. (**b**) data of the second left square in another region of the acceleration. (**c**) data of the square in the left part of the white paper. (**d**) data of the rightmost square in the central part of the white paper and covering a black strip.

Figure 4 shows an image strip (turned 90°counterclockwise with respect to those shown in Figure 2(b)) captured by properly adjusting the view of the high-speed camera. Figure 4 shows four squares: the two squares on the left side enclose different parts of the accelerometer and the other two contain locations of the white paper with and without the black straight line. Four typical strings of the gray-level data corresponding to the four different squares are separately plotted in Figure 5(a) through 5(d). The minimal and maximal gray-level sums of these squares are (1,166, 1,496), (29,638, 32,074), (121,823, 121,943), and (85,543, 97,420). These resolutions are extremely poor, poor, extremely poor, and good, respectively. The image of the leftmost square and its neighboring region is extremely dark so that the maximal gray-level variation is less than 330 gray levels, as shown in Figure 5(a). Similarly, the third left square and its neighboring region are extremely bright so that the gray-level summation has the worst capability of signal resolution, as shown in Figure 5(c). The second left square has relatively better image resolution than the two mentioned squares, but the maximal gray-level variation is not prominent. When the image contrast was artificially enhanced by the black straight line, the gray-level resolution rose to approximately 10,000, as shown in Figure 5(d). This means that the increase of the brightness contrast by the black line substantially improves the capability to resolve the vibration information.

### 2.4. Modeling Non-Constant Data Converting Ratio Effect of Insufficient Frame Rate

To look into the mechanism embedded in the image data, assume that an amount of the induced photo-charge at the time *t* in a photo-diode decays with a factor of exp[−(*t*′−*t*)/*τ*] at a subsequent instance *t*′, where *τ* is the averaged time constant of the charge attenuation [27,28]. This model directly simulates the effect of the afterimage. It also reflects the effect induced by the image lag because the corresponding factor 1 − exp[−(*t*′−*t*)/*τ*] can properly simulate the time delay. Using this simplified model, for a light ray with an intensity of sin2*πft* reaching a photo-diode, the accumulated photo-charge at the instant *t* is simulated by the following integration:

(2) $$\int_{t-\alpha }^{t}e^{-(t-x)/\tau }\sin2\pi \text{fxdx}=L(t)=\frac{1}{1+4\pi^{2}f^{2}\tau^{2}}\{[\tau \sin2\pi ft-2\pi \tau^{2}\cos2\pi ft]-e^{-\alpha /\tau }[\tau \sin2\pi f(t-\alpha )-2\pi f\tau^{2}\cos2\pi f(t-\alpha )]\}$$

where α is defined as the constant satisfies exp[–*α*/*τ*] = 0.05. Equation (2) shows that the afterimage and hence the image lag do not induce any sinusoidal mode whose frequency is different from *f*

It is well known that the performance of the readout circuit depends on the illumination intensity of the incident ray [12–14]. To overcome the negative effect of the dependence and obtain a good image brightness and sharpness within the desired frame speed, certain compromises were properly addressed in the design stage. However, when the frequency of a vibration signal cannot be properly resolved by the designed frame speed, the non-linear dependence between the output gray level and photo-charge starts to play a negative effect. Since the variation of a light intensity is too fast, the readout circuit ends up to pick a bright part and partially ignore the dark part. Therefore, as an initial study, the following simplified model of the gray level readout with respect to the photo-current *L*(*t*) of Equation (2) is proposed:

(3) $$g=g_{c}+k(1-e^{-[\beta -\omega L(t)]})L(t)$$

where *g* is the output gray level, *g_c_* is a reset constant and *k* is the proportional constant, *β* is an empirical discharge time constant, and the threshold factor *ω* is defined as:

(4) $$\begin{array}{ccc} \omega & =0, & f\leq f_{c}\\ & =1, & f>f_{c} \end{array}$$

in which *f_c_* is the critical frequency of inducing the non-physical response. Since our main concerning is the frequency identification that does not depend on the exact values of *g_c_* and *k*, they are set to be 0 and 1, respectively.

**Figure 6.**
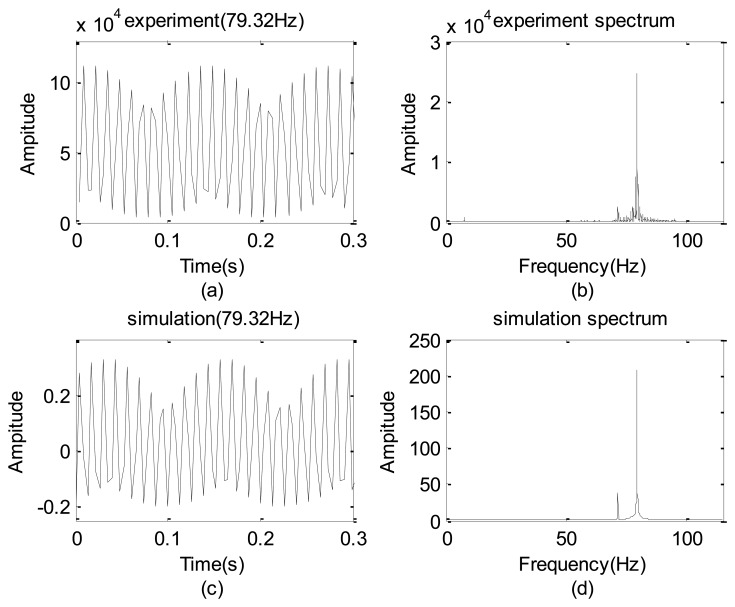
Comparison between experimental and modeled data: (**a**) data of the experiment; (**b**) spectrum of experiment; (**c**) data of simulation; and (**d**) spectrum of simulation.

To identify *β*, the LED light signal shown in Figure 1(a,b) were tested with a given frequency of 79.32 Hz. Figure 6(a) is the measured gray level sum taking by the high speed camera and Figure 6(b) shows the corresponding spectrum.

After a few cycles of try and error, *β* = 2.3 is chosen and the resulting and spectrum are shown in Figure 6(c,d), respectively. Clearly, Figure 6(c) is in some sense similar to Figure 6(a). A careful inspection on these spectra reveals that frequencies of their non-physical modes (also shown in Table 1) have a deviation of 0.01% so that the proposed simple model is reasonable. Thus, this model is used to predict the false modes in Section 4.

## 3. Tools of Data Analysis

Because the data detected using the mentioned data acquisition systems frequently drift, they may contain a monotonic non-periodic part. For convenience, these time series data strings are written in the following form [1,7–9,19]:

(5) $$\begin{array}{cc} y_{j}=\sum_{l=0}^{J-1}[b_{l}\cos\frac{2\pi t_{j}}{\lambda_{l}}+c_{l}\sin\frac{2\pi t_{j}}{\lambda_{l}}]+\sum_{n=0}^{N}a_{n}t_{j}^{n}, & 0<j<J \end{array}$$

where *y_j_* is the gray-level summation at *t* = *t_j_*, the second summation represents the non-sinusoidal trend, and *N* represents the largest power for which *a_n_*→0 for all *n* > *N*. In most engineering applications, *N* = 250 is a reasonable value. The non-sinusoidal trend is interpreted as the sum of monotonic parts and all of the Fourier modes, in which the wavelengths are longer than the data span *T* = *J*Δ*t*. The iterative Gaussian smoothing method in the spectral domain is used as a high-passed filter and yields the following high-frequency response [7–9,19–21]:

(6) $$y_{m}^{\prime }=\sum_{l=0}^{J-1}{\left[1-a(\sigma /\lambda_{l})\right]}^{m}\left[b_{l}\cos\left(\frac{2\pi t}{\lambda_{l}}\right)+c_{l}\sin\left(\frac{2\pi t}{\lambda_{l}}\right)\right]$$

where the iteration parameter *m* and smoothing factor *σ* use the values of 127 and 0.8*T*, respectively. Since *m* > 125 is corresponding to *N* > 250, the trend is ultimately removed, because this result is derived by assuming the data span running from –∞ to ∞, small errors are thus induced by using the missing data beyond the two ends. Next, the zero crossing points around the two ends can be located by performing a search procedure and by implementing an interpolation formula. After dropping the data segments beyond the two zeros, a monotonic interpolation [29] is used to redistribute the data into uniform spacing in which the points equal an integer power of 2. Subsequently, an odd function mapping is used to double the data span. Finally, the fast Fourier transform (FFT) [22] generates a discrete Fourier sine spectrum. This spectrum can reflect many details embedded in *y*(*t*) because the associated FFT is mathematically a complete expansion, such that the trend has already been removed and all of the required periodic conditions are ensured by using the odd function mapping.

In this study, the following Gabor wavelet transform using the Gaussian window (with window width *a*) is used for a sinusoidal data *y*(*t*) [1,19–21,23–26].

(7) $$G(f,\tau )=\frac{1}{\sqrt{a}}\int_{0}^{T}y(t)e^{-2i\pi f(t-\tau )}e^{-{\left(t-\tau \right)}^{2}/(2a^{2})}dt$$

That it is approximately equal to the following form can be proven [7–9,19]:

(8) $$G(f,\tau )\approx \sqrt{\frac{a\pi }{2}}\sum_{l=1}^{J-1}\left\{[c_{l}-id_{l}]e^{i2\pi f_{l}\tau }\exp[-2a^{2}\pi^{2}{\left(f_{l}-f\right)}^{2}]\right\}$$

These relationships indicate that the wavelet coefficient is only an inverse FFT of a finite spectrum band specified by using an associated Gaussian window. The resulting wavelet coefficients are subject to the blur effect of the uncertainty principle [23–26].

## 4. Results and Discussion

**Figure 7.**
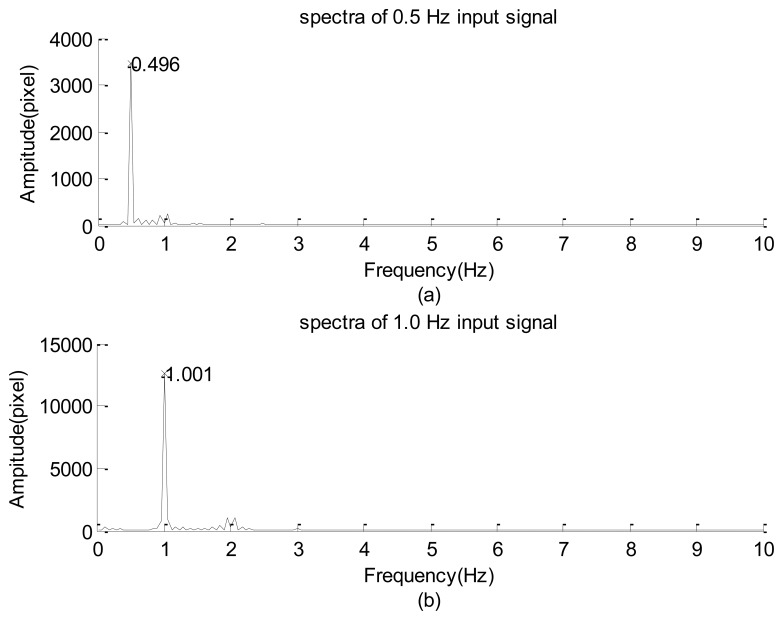
Spectra distribution of LED signal collected by the high speed camera: (**a**) 0.5 Hz input signal. (**b**) 1 Hz input signal.

The experimental design of Figure 1(a) was first used to examine the frequency response of the high-speed camera with the image signal emitted from a green LED. The power of emanating the light was supported using a function generator. The input signal was a sine wave with 1-V amplitude in addition to a direct current of 2 V. During the period of data collection, all of the other illumination light sources were turned off so that all of the image frames were similar to that shown in Figure 1(b). The solid lines of Figure 3(a,b) indicate the 0.5-Hz and 1.0-Hz raw data of the gray-level summation of all of the pixels within the square of Figure 1(b). The dashed line is the smooth part decoupled by the iterative Gaussian filter with *σ* = 2.5 s and 127 iterations. The corresponding Fourier sine spectra of the high-frequency part are depicted in Figure 7(a,b), respectively. Because inputs of the function generator were controlled by turning a circular knob manually, the frequency readings of the two input modes of Figure 7(a,b), 0.496 and 1.001 Hz, are considered the corresponding exact input frequencies.

Figure 7(a,b) show that the amplitudes of their dominant modes are larger than the corresponding maximal amplitude among all of the minor modes by more than one order of magnitude. Therefore, the high-speed camera successfully captured the main information embedded in the emitted light of the LED.

**Figure 8.**
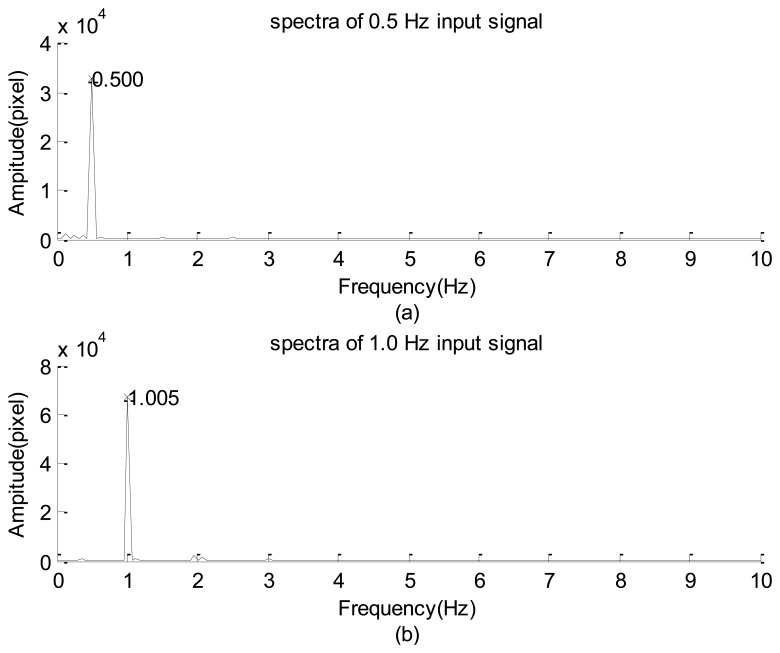
Spectra distribution of LED signal collected by the webcam: (**a**) 0.5 Hz input signal. (**b**) 1.0 Hz input signal.

After replacing the high-speed camera with the webcam, the resulting spectra of the LED signals shown in Figure 8(a,b) are results of using input frequencies of approximately 0.500 and 1.005 Hz. A careful inspection of these figures reveals that the main features of Figure 8(a,b) are similar to those of Figure 7(a,b), respectively. In general, these results show that the image data acquisitions using the webcam and high-speed camera are reliable for capturing the sinusoidal signals of LED light. In addition, the scattered dominant and minor modes in these spectra, combined with the non-symmetric distributions of the high-frequency part shown in Figures 3(a) and 7(b), show that the relationship between the illumination of the LED and the input power is non-linear.

Table 1 compares several dominant non-physical modes of both experimental and modeled results, in which all of the error are reasonably small. Moreover, non-physical mode's frequency decays as the given frequency is increased whose mechanism needs further study. Nevertheless, all of the errors are reasonably small which shows that the simplified model is acceptable. A series of tests show that, when the LED signal was examined by the webcam, the latter automatically changes its sampling rate because the illumination intensity is too low. Although the webcam data also confirm the validity of the proposed model, therefore, the webcam data is not shown here.

**Figure 9.**
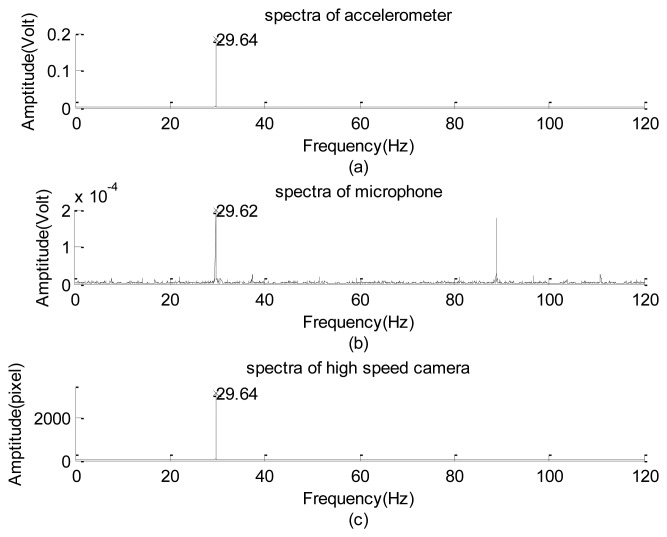
Comparison of the frequency response of the vibration exciter's signal, input frequency is about 30 Hz: (**a**) spectra of accelerometer. (**b**) spectra of microphone. (**c**) spectra of high speed camera.

Next, the experimental design of Figure 2 was used to test the frequency response of the image signal of the high-speed camera. Frequency responses of the high-frequency part of the data collected using the accelerometer, microphone, and high-speed camera are plotted in Figure 9(a) through 9(c), respectively. Their corresponding raw data are not shown because of the length limitation. Again, the input frequency can only be roughly recognized as 30 ± 1.0 Hz. Therefore, the exact input frequency using the accelerometer was identified at 29.64 Hz, as shown in Figure 9(a).

The amplitude of the vertical vibration of the black line (with an effective thickness approximately equaling 1 pixel on every image frame) is 0.536 ± 0.010 mm, as measured using a dogmatic caliper. Frequencies of the input mode captured using the microphone and high-speed camera are 29.62 and 29.64 Hz, respectively, which slightly deviate from the reading of the accelerometer data. The microphone data can only be considered a reference here because it also collected the acoustic information generated by the motion of the rubber membrane. In addition to the input modes, the image system reflected the first harmonic and minor modes of 83 Hz, which can be roughly explained as the composite mode of the input, first harmonic, and the sub-harmonic modes. Compared with the amplitude of the input mode (≈3,166 gray levels), the first harmonic and the minor modes are approximately 1.8% and 1.4%, respectively. They are too small to be made visible in Figure 9(c) using the drawing program of the Matlab software [30]. Therefore, the frequency response of the high-speed camera is consistent with that of the accelerometer. A similar performance of the camera was found when the input frequency was less than 60 Hz. However, when the frequency was equal to or greater than 60 Hz, the non-physical mode generated by the camera began to play a certain role.

**Figure 10.**
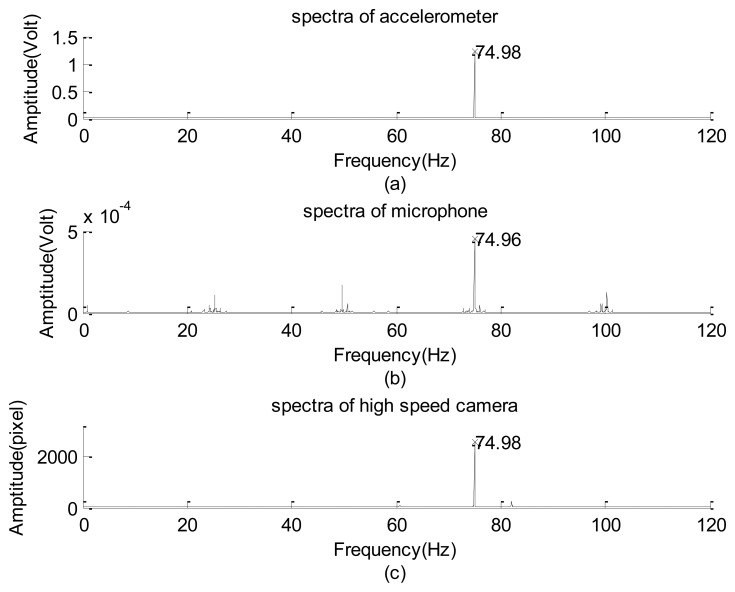
Comparison of the frequency response of the vibration exciter's signal, input frequency is about 75 Hz: (**a**) spectra of accelerometer. (**b**) spectra of microphone. (**c**) spectra of high speed camera.

Figure 10(a) through 10(c) respectively show the spectra detected using the accelerometer, microphone, and high-speed camera with an input frequency of 75 Hz. The input mode is resolved using the three sensors as 74.98 ± 0.01 Hz. The other significant sub-harmonics and harmonics captured by using the microphone are not shown in Figure 10(a) or 10(c); therefore, they are acoustic signals. Regarding the image data, the 61 and 82 Hz modes are not shown in Figure 10(a) or 10(b). Their corresponding amplitudes are 64 and 249 gray levels, respectively. Compared with the input mode in which the amplitude is 2,577, the 61 Hz mode can also be interpreted as the 4/5 sub-harmonic mode and may be ignored, but the 82 Hz mode cannot. Because it cannot be properly explained, this mode is referred to as “non-physical mode”. A series of tests were completed to identify the critical frequency of the input signal that induces the false response.

Table 2 lists several results of the input mode of the image signal, the input frequency, and a few significant minor modes. The input frequencies were read from the spectrum of the accelerometer data. Those listed in the remaining columns are the 100% amplitudes with respect to the amplitude of the input mode. Several modes marked with the “plus” sign were simultaneously captured using the high-speed camera and microphone, meaning that they are physical modes. Those frequencies marked with “underline” sign are approximately interpreted as the sub-harmonics, harmonics or combinations between sub-harmonic and harmonic. Except the 83.84 and 92.72 Hz modes (using input frequency of 29.64 and 79.54 Hz, respectively) whose mechanisms are not known yet, all of the rest of minor modes without the plus sign are referred to as non-physical modes because they cannot be properly explained regarding the mechanism of the vibration exciter.

**Figure 11.**
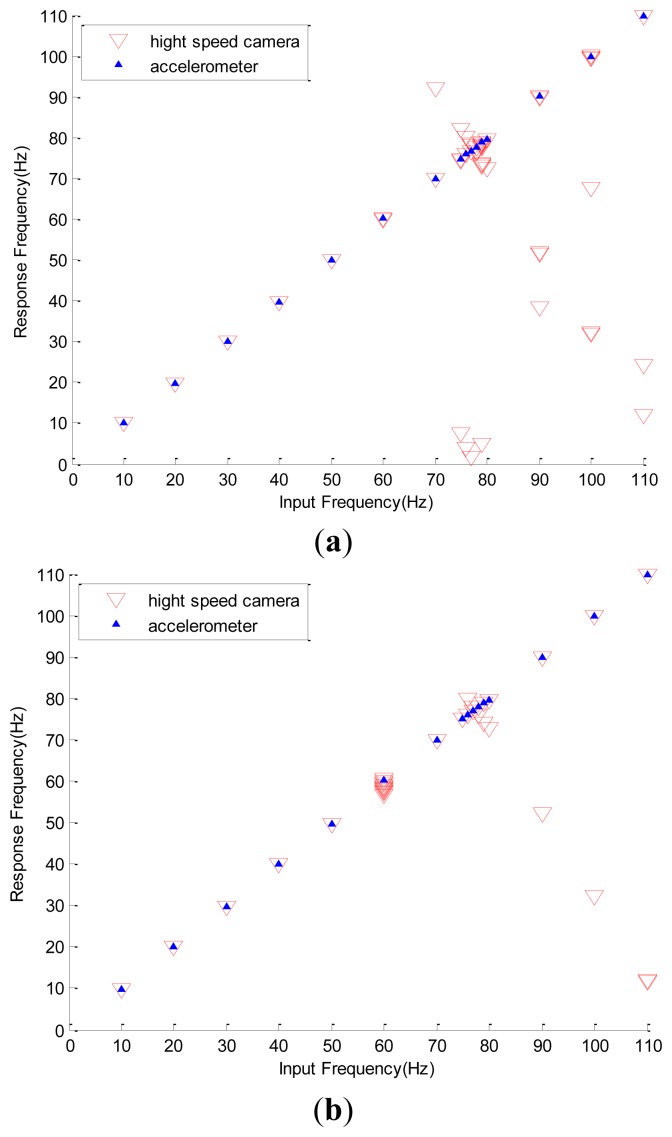
Significant modes captured by the high speed camera and accelerometer: (**a**) the amplitude of the exciter's vibration is 0.536 mm. (**b**) the amplitude of exciter's vibration is 0.371 mm.

In Table 3, deviations of the simulated frequencies from the corresponding experimental data are listed. It is interesting to see that all the frequency differences between the image and simulated data are less than 0.2%. For the sake of clarity, all of the data are also plotted in Figure 11(a), where the horizontal axis is the accelerometer reading and the vertical axis represents the input and minor modes reflected by the high-speed camera. All minor modes in which the amplitudes are less than 10% of that of the input mode are not shown. Figure 11(b) also shows the similar frequency responses with the vibration amplitude of the exciter adopting a value of 0.371 mm. Figure 11(a) shows that the non-physical mode is not seen in the region where the input frequency is less than 60 Hz. The detailed data also show that, as the input frequency is less than 60 Hz, all of the percentage amplitudes of the non-physical modes are less than 1.5%. When the input frequency is 60 Hz, many delta symbols emerge. Similarly, Figure 11(b) shows that the false modes are found when the input frequency is large and equal to 60 Hz. In other words, the large error of the 60 Hz mode in Table 2 and the clustered delta symbols around the position of 60 Hz in Figure 11(a) are induced by insufficient frame rate.

All of the previous discussions indicate that the Prosilica CV640C high-speed camera demonstrates significant non-physical response when the image vibration frequency is larger or equal to 60 Hz. Thus, the recharge time constant of the readout circuit of the high-speed camera is approximately 1/60 s. Therefore, if the high-speed camera is used without the support of other sensors, a precise spectrum may not be obtained when the input frequency is larger than and/or equal to 60 Hz. Fortunately, all of the non-physical modes can be predicted by the proposed simple model with small error. Thus they can be properly excluded from the spectrum. Moreover, except for the 60 Hz mode, Table 2 and Figure 11(a,b) show that all of the differences of all of the dominant modes between the accelerometer data and image data are less than 0.02 Hz. This means that, after excluding the false modes, the physical signals are faithfully reflected by the image sensor.

**Figure 12.**
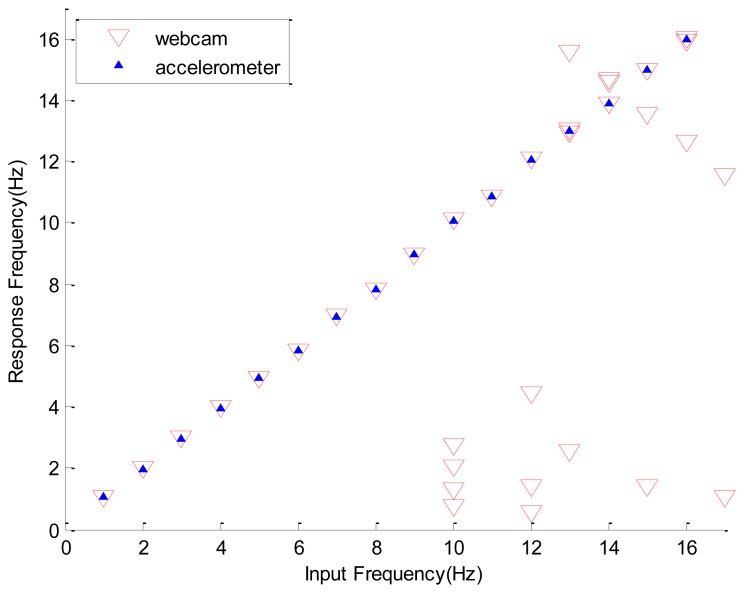
Significant mode captured by webcam and accelerometer, the amplitude of exciter's vibration is 0.536 mm.

Following a similar procedure, the frequency responses of the webcam with a sampling rate of 30 FPS and the accelerometer with the sinusoidal amplitude of the exciter being 0.536 mm are shown in Figure 12. Again, the image data obtained by the webcam precisely reflect the input frequency read by the accelerometer. Detailed deviations of the simulated frequencies from the corresponding experimental data are listed in Table 4. Now the maximal simulation error is less than 3% and the non-physical response is induced when the input frequency is equal to or larger than 7.8 Hz.

In addition to the input amplitude and frequency, the spectrum and non-physical response modes of the obtained image data also depend on several factors: the line thickness marked on the white paper sheet, intensity of the illuminating light, and the shutter speed of the camera. For convenience, we used the high-speed camera and fixed input frequency of the vibration exciter to demonstrate effects of varying these factors.

Tables 5–7 list the dominant mode and a few non-physical modes of changing one of these parameters. The signals shown in Table 5 were obtained using the high-speed camera with five effective image thicknesses (1, 3, 5, 7, and 9 pixels) of the black line, combined with a fixed input frequency of approximately 80 Hz. As the line thickness increases, amplitudes of the resulting false mode monotonically decrease. For example, the 72 Hz false mode of the case with a 1-pixel thickness has the relative amplitude of 110% with respect to the dominant mode; that of the 2-pixel case is 50%, and that of the 9-pixel case is 14%. This is reasonable because a small degree of vertical vibration of a thin black line can easily induce the variation of the light intensity reaching the photodiode that eventually leads to significant false response. Conversely, a thick black line with the same amplitude of vertical vibration may not generate a significant gray-level variation of the image data; hence, it is not easy to trigger the non-physical response.

Table 6 lists the results of using the input frequency of 80 Hz with several different light intensities reaching the white sheet of paper. A rough inspection reveals that an optimal illumination exists, in which the 404-LUX case introduces the minimal amplitude of the false mode with respect to all of the other illumination cases. Table 7 lists the input and non-physical modes captured using the high-speed camera with the 90 Hz input frequency for several shutter speeds. Again, for the purpose of suppressing the false response, the data reflect that an optimal shutter speed exists for the suppression at approximately 1/1,000 s. These tables indicate that one of the three factors can be properly changed to partially suppress the negative effect of the false response. When more than one of these factors is tuned, the non-physical mode cannot be completely eliminated (results not shown).

In general, all commercial webcams are equipped with functions for automatically adjusting the light exposure value and shutter speed. Consequently, a user cannot easily adjust these two parameters. Therefore, we merely examined the effect of varying the thickness of the dark line when generating non-physical information with and without an additional light source. For convenience, the input frequency of the vibration exciter was set at approximately 13 Hz. Table 8 lists the input and several main non-physical modes captured by the webcam without switching on the car lamp. Results of switching on the light source of the car lamp are tabulated in Table 9. These two tables show that there is no optimal line thickness for the reduction of the amplitude of the false mode. All of the mentioned discussions and other results not presented here show that, once the vibration modes are larger than the critical frequency the non-physical mode cannot be effectively eliminated by changing one or more available factors.

**Figure 13.**
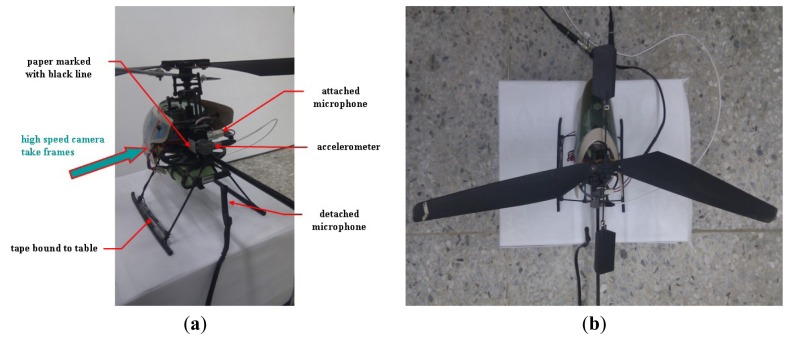
The experimental setup of capturing the signal of a small electric helicopter: (**a**) the overall set up. (**b**) the non-symmetrically main rotor wing.

Finally, two practical applications of employing the image sensors to detect the unsteady signals are discussed. The first involved using the high-speed camera and microphone to capture the vibration signal of a small electrical helicopter bound to a table (see Figure 13(a)). Because the operation signal of the helicopter was too small to be captured using the accelerometer, the blades on both sides of the main rotor wing were non-symmetrically reset, as shown in Figure 13(b), to enlarge the degree of vibration. The image system can properly reflect the vibration signal of the original helicopter [8,9] and is not discussed here.

**Figure 14.**
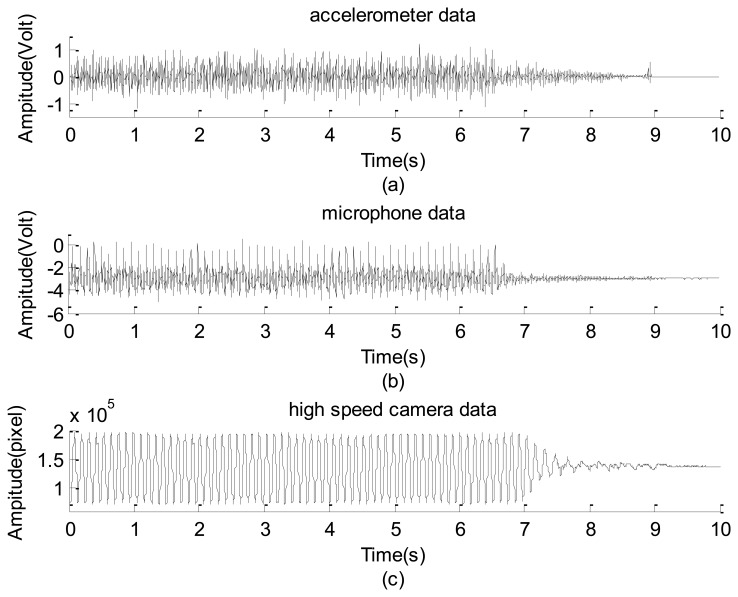
The raw data of the small helicopter in operation captured by: (**a**) accelerometer. (**b**) microphone. (**c**) high speed camera.

During the test period, the accelerometer and a microphone were attached to the body of the helicopter, and a detached microphone was near the tail bar of the helicopter. Before collecting the signal, the helicopter was first powered on until it reached the steady state. After collecting the data for 7 s, the power was turned off. Because the detached microphone could not provide a useful vibration signal, its data are not discussed. Those shown in Figure 14(a) through 14(c) are the raw data collected using the accelerometer, the attached microphone, and high-speed camera, respectively. These data cannot reflect much information except that their envelopes consistently reflect the attenuation after switching off the power.

**Figure 15.**
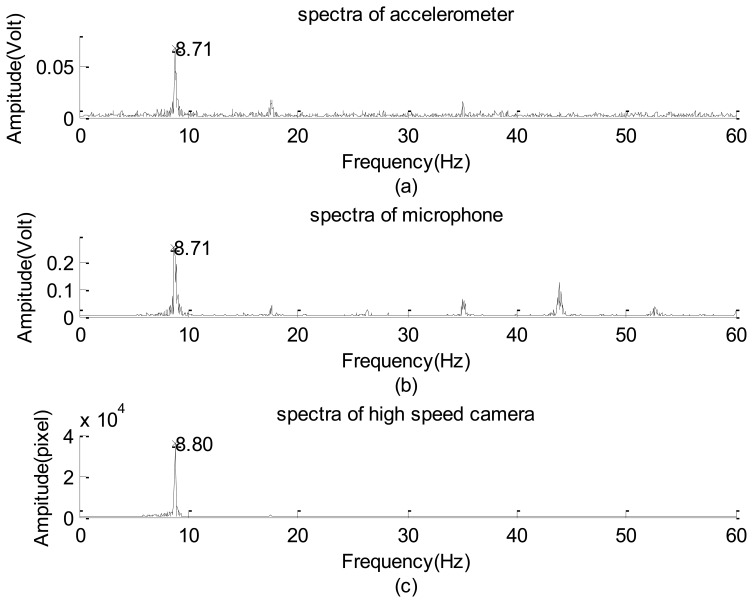
Spectra of the small helicopter in operation captured by: (**a**) accelerometer. (**b**) microphone. (**c**) high speed camera.

Their corresponding spectra and wavelet spectrograms are shown in Figures 15(a) through 15(c) and 16(a) through 16(c), respectively. They show that the dominant (9 Hz) and several harmonics are consistent with one another. Because the test vibration signal was still too weak to be clearly captured using the accelerometer, in addition to the dominant mode, only the first and third harmonics are prominently reflected. Therefore, its spectrogram involves many random and scattered noises. Nevertheless, both the microphone and high-speed camera clearly captured the dominant and many harmonics. The amplitudes of the first, third, fourth, and fifth harmonics of the microphone data are in the same order of the dominant mode, meaning that they are dominated by the aero-acoustic signal rather than the vibration signal. However, the corresponding harmonics of the image signal are extremely small so that they are purely vibration signals. This is reasonable because the acoustic signal of a flow field is invisible with respect to an image system. These charts show that most vibration modes emitted by all components of the helicopter are insignificant as compared with that of the fundamental mode of the unbalanced main rotor. Because the random noise of the high-speed camera is also extremely small, we conclude that the helicopter was in a functional condition when it was tested. Furthermore, based on the spectrograms of the image signal, the frequency of the main rotor linearly decayed in the power-off period. However, the microphone could not capture the same datum because the acoustic signal of the rotor was rapidly dissipated by the flow field to an insignificant level.

**Figure 17.**
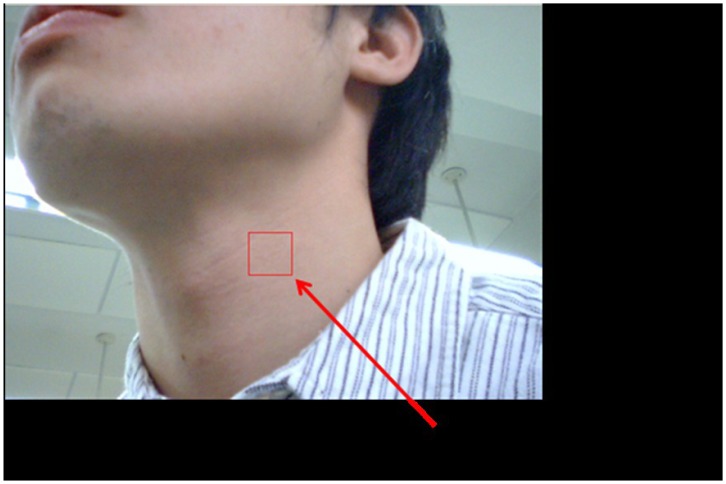
The experimental setup for measuring the neck arterial pressure signal via a webcam and the finger blood-oxygen saturation level.

The second practical application involved using the webcam to obtain the neck arterial pressure signal of a young man (see Figure 17). The man statically sat on a chair to avoid the requirement of dynamically monitoring his neck's motion. Since he was not allowed to move forward and/or backward, the square covered almost a fixed region so that the captured signal was not dominated by the information introduced by his motion. Simultaneously, the person's left forefinger was mounted using an HYAYU pulse oximeter to collect his oxygen saturation signal. The oximeter has a sampling rate of 200 Hz/24 b and provides ±3% error of detections for the heartbeat frequency and the level of the blood-oxygen saturation.

**Figure 18.**
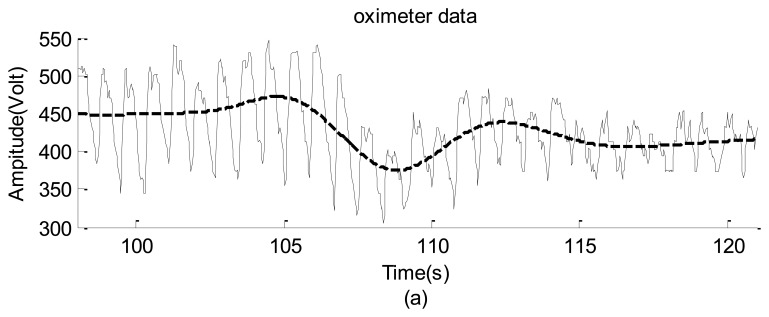

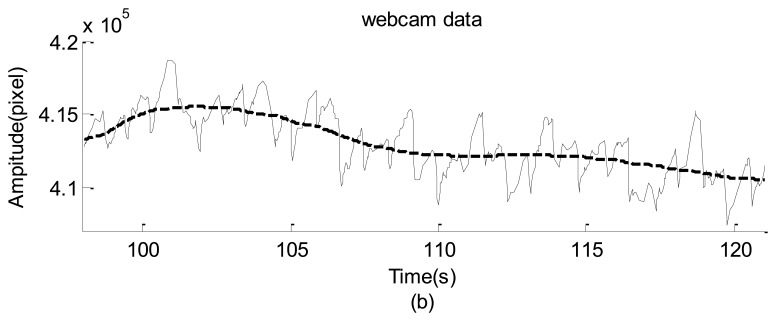
The raw data of the neck arterial pressure signal of an healthy adult: (**a**) the level of the blood-oxygen saturation captured by the pulse oximeter; and (**b**) captured by the high speed camera.

**Figure 19.**
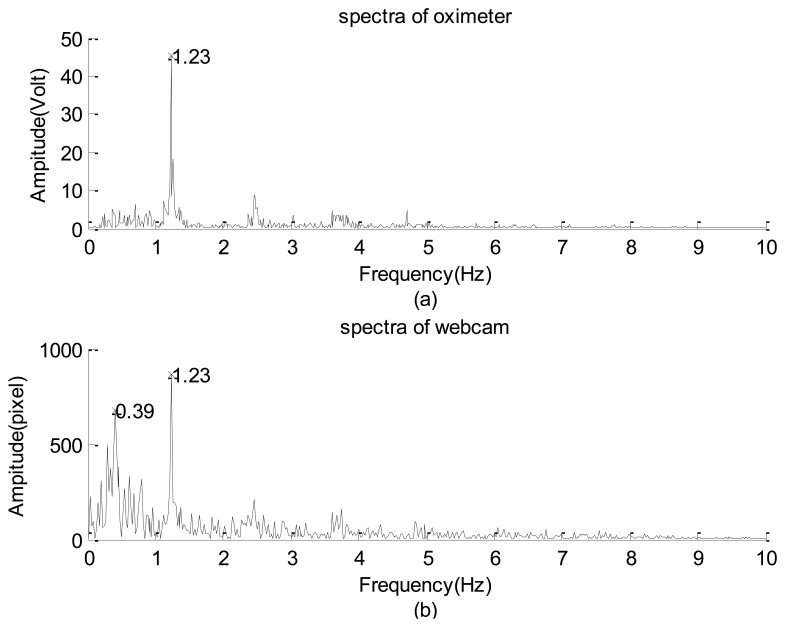
Spectra of the neck arterial pressure signal of an healthy adult: (**a**) the level of the blood-oxygen saturation captured by the pulse oximeter. (**b**) captured by the webcam.

**Figure 20.**
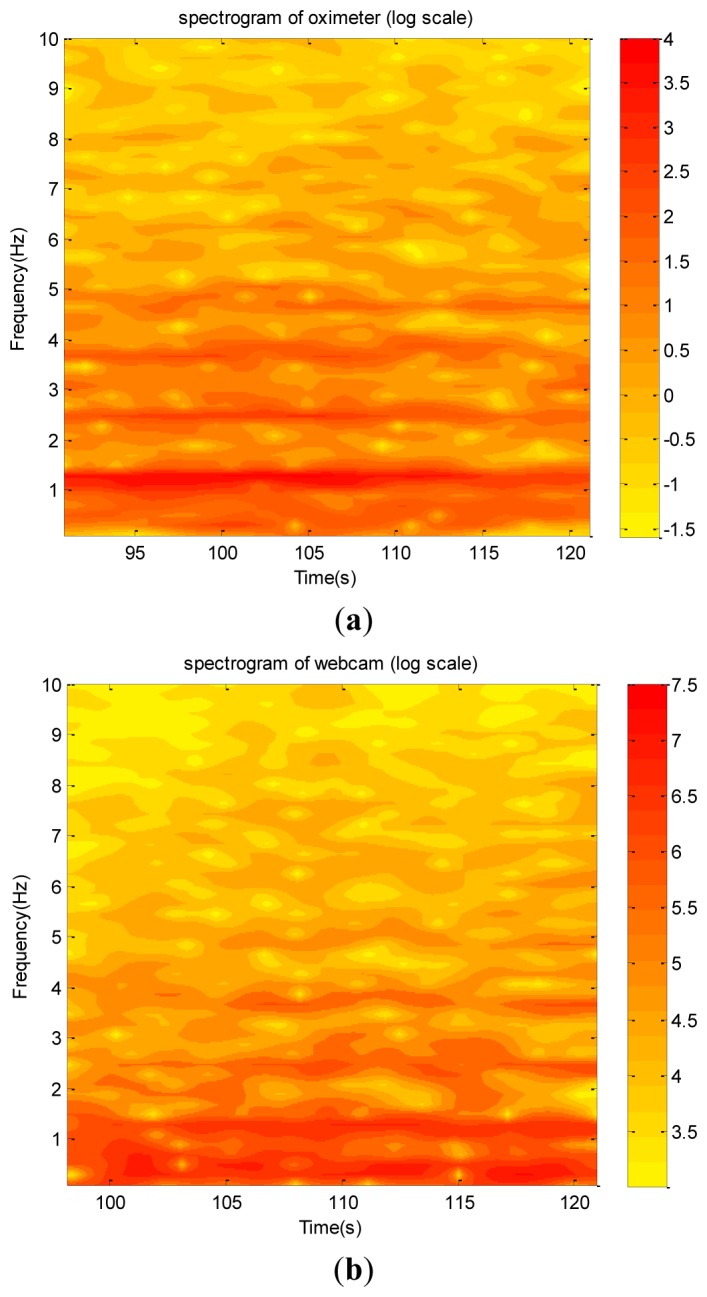
The wavelet coefficient plots of the neck arterial pressure signal of an healthy adult: (**a**) the level of the blood-oxygen saturation captured by the pulse oximeter. (**b**) captured by the webcam.

The raw data are shown in Figure 18(a,b), which do not provide any detailed information. The corresponding spectra and spectrograms are shown in Figures 19(a,b) and 20(a,b), respectively. These figures involve many minor modes and noises that cannot be properly explained because no other data measured using any sophisticated instrument were available. Nevertheless, based on both spectra, the heartbeat mode (1.23 Hz) can be easily identified, and the difference between them is less than 0.1 Hz. This means that the webcam accurately obtained the most critical mode of a human being. In addition, the image data clearly reflect the prominent breathing mode (0.39 Hz), but the oximeter could not clearly obtain the mode, meaning that the variation of the blood oxygen data of the forefinger is not closely related to the human breathing mode. Based on both spectrograms, the amplitudes and frequencies of the dominant mode demonstrate significant fluctuations. This indicates that the respiratory and circulatory systems of the adult human have high degrees of adaptation. In other words, the adult was in a healthy state at the instant of taking data.

Finally, the previous discussions are summarized into the following facts:

1. When all prominent modes of a vibration signal are less than the critical frequency of generating the false mode, the dominant and harmonics captured using the high-speed camera and webcam are consistent with those reflected by the accelerometer and microphone.
2. The image data may be contaminated by the non-physical response induced by the insufficient frame rate of the camera. When a vibration signal involves one or more significant modes in which the frequencies are higher than the critical frequency, the non-physical modes are generated. The critical frequencies of the high-speed camera and webcam are 60 and 10 Hz, respectively. These false modes cannot be effectively excluded by changing the incident light intensity or the image signal-enhancing techniques.
3. A simple model is proposed to accurately predict the non-physical modes. Consequently, all the non-physical modes can be identified and excluded from the spectrum.
4. The two practical tests show that the image data acquisition system has the potential to capture miniscule vibration signals consistently in a non-contact manner.

## 5. Conclusions

Experimental results show that both the high-speed camera and webcam precisely capture the dominant and harmonic modes. Although the spectra may be blurred by the non-physical response when the frequencies of the significant vibration modes are equal to or larger than a critical frequency, most of the false modes can be identified and effectively excluded by a simple model. The critical frequencies of induction of the non-physical response of the high-speed camera and webcam, which are each induced by the insufficient recharge time of the pixel circuits of the image sensor, are different. The proposed system is simple, convenient, and precise and can now be considered a remote data acquisition system to evaluate the vibration spectrum of a system in operation.

## Figures and Tables

**Figure 16.**
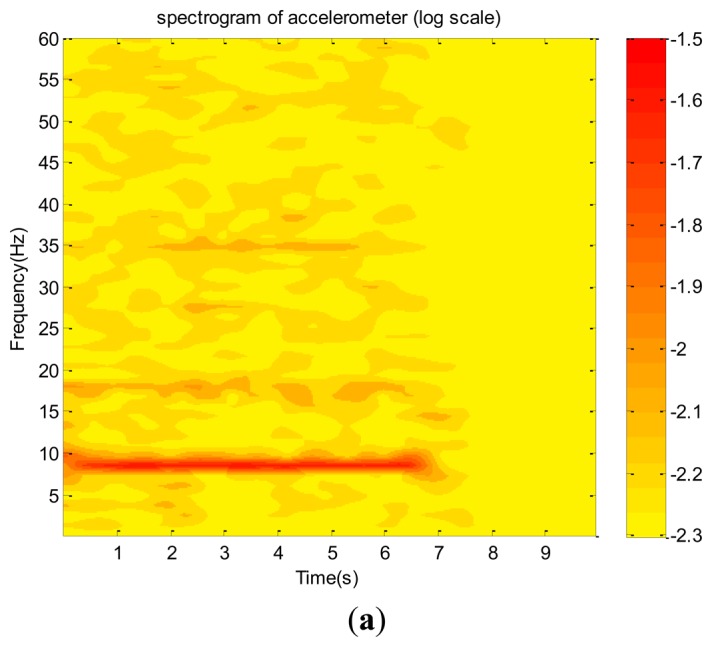

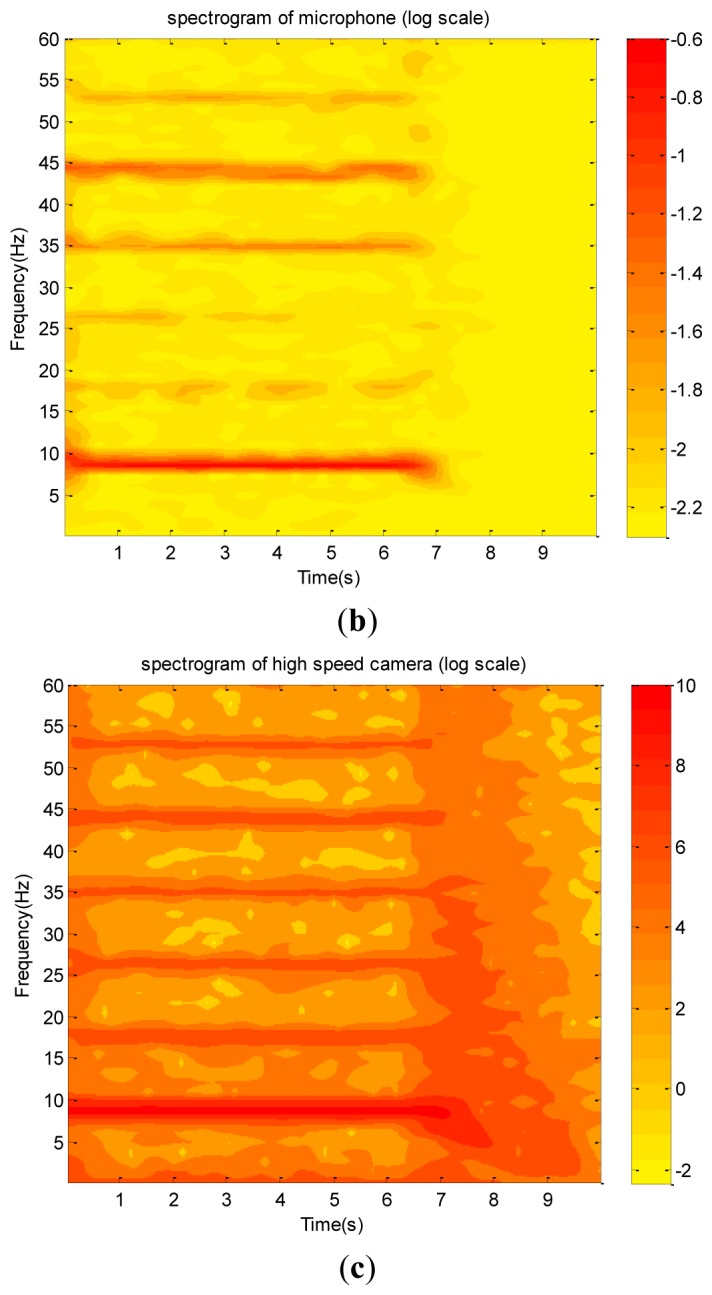
The wavelet coefficient plots log scale of the small helicopter in operation captured by: (**a**) accelerometer. (**b**) microphone. (**c**) high speed camera.

**Table 1.** Frequency Comparison of the Dominant Non-Physical Mode, Using High Speed Camera to Capture LED signal.

**Input Hz**	**Dominant physical modes (Hz)**	

	**Experiment**	**Error**
69.91	91.97	−0.08%
79.32	71.49	0.01%
89.99	52.04	−0.08%
99.62	32.84	−0.12%

**Table 2.** Frequency response of the high speed camera.

**Dominant mode**		**Input Hz** *	**Minor modes**							
	
**Hz**	**amp.**		**Hz**	**amp** ^!^	**Hz**	**amp** ^!^	**Hz**	**amp** ^!^	**Hz**	**amp** ^!^

9.97	3605	9.97	19.93	0.7%	42.68	0.6%	62.62	1.0%	69.76	1.2%
20.11	3013	20.11	40.27	1.0%	51.03	1.3%	60.33	1.4%	80.49	0.7%
29.64	3166	29.64	59.33	1.8%	83.84	1.4%				
40.06	3246	40.06	80.17	2.5%						
49.79	3018	49.78	99.52	2.8%						
58.56 ^#^	1675	60.21	108.04 ^+^	5.3%						
69.96	2684	69.96	25.68	2.7%	92.13	7.9%	114.25 ^+^	3.8%		
74.98	2577	74.98	60.79	2.5%	67.86	2.1%	82.10	9.6%		
76.02	2463	76.02	68.10	3.7%	72.06	2.6%	79.99	14.5%		
78.02	2569	78.02	73.96	2.6%	75.96	12.0%	80.08 ^+^	3.2%	82.08	2.7%
79.54	2481	79.53	72.92	11.2%	92.72	3.2%				
89.92	2290	89.92	37.77	2.5%	52.16	19.3%	113.20 ^+^	2.1%		

* Input frequency measured by accelerometer.

! % relative amplitude of other significant modes with respect to the dominant mode.

+ The mode also captured by the microphone.

# Many modes whose frequency near 60 Hz are captured.

**Table 3.** Frequency Comparison of the Dominant Non-Physical Mode, Using High Speed Camera to Capture Vibration Exciter Signal.

**Input Hz**	**Dominant non-physical modes**			

	**Hz**	**Error**	**Hz**	**Error**

69.96	92.13	−0.03%		
74.98	67.86	−0.06%	82.10	0.00%
76.02	72.06	+0.19%	79.99	+0.01%
77.03	77.98	+0.03%		
78.02	75.96	+0.05%		
78.99	74.08	+0.03%		
79.54	72.92	+0.11%	86.11	+0.10%
89.93	37.77	+0.08%	52.16	+0.08%
99.87	32.24	+0.19%	67.58	+0.03%

**Table 4.** Frequency Comparison of the Dominant Non-Physical Mode, Using Webcam to Capture Vibration Exciter Signal.

**Input Hz**	**Dominant non-physical modes (Hz)**	

	**Experiment**	**Error**

7.81	13.01	−0.08%
8.96	10.60	+0.94%
10.07	8.43	−0.45%
10.86	6.83	+1.04%
12.09	4.39	+0.23%
13.06	2.57	−2.72%

**Table 5.** Frequency response of the high speed camera with different black line thickness.

**Dominant mode**		**Pixel**	**Other modes**							
**Hz**	**amp.**		**Hz**	**amp** ^!^	**Hz**	**amp** ^!^	**Hz**	**amp** ^!^	**Hz**	**amp** ^!^
80.00	851	1	7.87	15.3%	23.57	8.1%	39.32	10.3%	72.07	109.8%
80.08	2,934	3	8.17	5.3%	24.67	1.0%	39.01	3.3%	71.86	50.0%
80.45	5,239	5	9.38	10.4%	28.09	3.9%	42.99	3.5%	71.12	35.4%
79.99	7,515	7					40.27	0.9%	72.01	17.0%
79.92	11,746	9	7.72	2.4%	23.16	0.9%			72.15	14.0%

**Table 6.** Frequency response of the high speed camera using different illumination of light source.

**Dominant mode**		**LUX**	**Other modes**					
	
**Hz**	**amp.**		**Hz**	**amp** ^!^	**Hz**	**amp** ^!^	**Hz**	**amp** ^!^

79.80	991	109			72.47	18.1%	87.12	12.3%
79.80	1930	206	7.38	5.2%	72.42	2.6%		
79.81	3436	304			72.39	4.4%		
79.84	4106	409			72.37	1.2%		
79.82	3747	505			72.35	17.8%		
79.85	2712	607	7.47	11.2%	72.33	73.3%	87.33	13.2%
79.83	2041	704	7.52	40.0%	72.46	220.9%	87.35	34.1%
79.84	1837	804	7.57	52.4%	72.32	261.2%	87.41	36.8%

**Table 7.** Frequency response of the high speed camera with different shutter speed.

**Dominant mode**		**Shutter speed**	**Other modes**					
	
**Hz**	**amp.**		**Hz**	**amp** ^!^	**Hz**	**amp** ^!^	**Hz**	**amp** ^!^

89.93	651	400	8.22	13.9%	14.50	8.6%		
89.89	892	600	8.23	18.2%	14.35	9.6%	52.27	3.5%
89.00	738	800	8.23	10.4%	14.35	11.5%	51.72	2.1%
89.91	1842	1000	8.22	2.2%	14.53	3.5%	52.17	6.9%
89.57	1175	1200	8.27	4.6%			52.00	13.7%
89.93	2477	1400	8.23	5.1%			52.21	8.0%
88.58	13,967	1600	8.23	5.6%			51.52	7.7%
89.88	2595	1800	5.82	1.3%			52.21	20.5%
89.52	1771	2000					52.14	53.3%

**Table 8.** Frequency response of the webcam with different black line thickness without enhancing light source.

**Dominant mode**		**Pixel**	**Other modes**					
	
**Hz**	**amp.**		**Hz**	**amp** ^!^	**Hz**	**amp** ^!^	**Hz**	**amp** ^!^

12.94	478	1	2.81	19.9%	5.82	10.6%	9.00	10.7%
12.87	1339	3	2.87	39.9%	5.69	9.6%	8.97	15.7%
12.85	2456	5	2.87	55.7%	5.74	15.3%	8.97	14.0%
12.85	3784	7	2.89	58.8%	5.71	9.0%	8.98	15.6%
12.84	4937	9	2.88	72.8%	5.80	8.4%	8.93	14.2%

**Table 9.** Frequency response of the webcam with different black line thickness with enhancing light source.

**Dominant mode**		**Pixel**	**Other modes**			
	
**Hz**	**amp.**		**Hz**	**amp** ^!^	**Hz**	**amp** ^!^

12.90	730	1	2.76	35.9%	9.01	11.7%
12.87	2180	3	2.81	21.8%	8.99	9.3%
12.88	3784	5	2.82	16.2%	9.03	9.3%
12.80	3347	7	2.89	22.3%	8.93	15.7%
12.85	6713	9	2.88	12.4%	9.00	11.6%
